# Multiscale Coupling of Transcranial Direct Current Stimulation to Neuron Electrodynamics: Modeling the Influence of the Transcranial Electric Field on Neuronal Depolarization

**DOI:** 10.1155/2014/360179

**Published:** 2014-10-23

**Authors:** Edward T. Dougherty, James C. Turner, Frank Vogel

**Affiliations:** ^1^Genetics, Bioinformatics, and Computational Biology Program, Virginia Polytechnic Institute and State University, Blacksburg, VA 24061, USA; ^2^Mathematics Department, Virginia Polytechnic Institute and State University, Blacksburg, VA 24061, USA; ^3^inuTech GmbH, Fuerther Straße 212, 90429 Nuremberg, Germany

## Abstract

Transcranial direct current stimulation (tDCS) continues to demonstrate success as a medical intervention for neurodegenerative diseases, psychological conditions, and traumatic brain injury recovery. One aspect of tDCS still not fully comprehended is the influence of the tDCS electric field on neural functionality. To address this issue, we present a mathematical, multiscale model that couples tDCS administration to neuron electrodynamics. We demonstrate the model's validity and medical applicability with computational simulations using an idealized two-dimensional domain and then an MRI-derived, three-dimensional human head geometry possessing inhomogeneous and anisotropic tissue conductivities. We exemplify the capabilities of these simulations with real-world tDCS electrode configurations and treatment parameters and compare the model's predictions to those attained from medical research studies. The model is implemented using efficient numerical strategies and solution techniques to allow the use of fine computational grids needed by the medical community.

## 1. Introduction

Transcranial direct current stimulation (tDCS) is a medical procedure that delivers electrical stimulation to the brain through electrodes positioned on the scalp. The electrodes deliver an electrical current on the order of 1-2 mA and produce an electric field in the patient's cerebral cavity that alters neuron excitability. A common use of this treatment is to assist neurons in firing action potentials (APs) by increasing their resting membrane potential. Current biomedical research continues to demonstrate the benefits of tDCS as a medical treatment. Recognition memory in Alzheimer disease patients has improved [1, 2]. Individuals who suffer from Parkinson's disease have demonstrated enhanced physical and mental skills [3, 4]. Patients diagnosed with neuropsychiatric disorders, including great depression, have shown improved cognitive capabilities [5, 6]. Further, poststroke recovery can be expedited with strategic administrations of tDCS [7, 8].

Current components of tDCS include the Laplace equation [9–13], which is given by

(1) $$\begin{array}{c} \nabla \cdot M\nabla \Phi =0,\;\vec{x}\in \Omega , \end{array}$$

where Φ is the electric potential, **M** represents the tissue conductivity tensor field, and *Ω* is the entire head cavity, including the brain. In isotropic tissues, **M** can be represented as a scalar which will vary among different tissue types.

Computational simulations of tDCS that utilize (1) have the capability to compute the strength of the tDCS electric potential and electric current at specific points in the brain and head cavity [11, 14]. What these models do not have, however, is the capability to provide a description of cellular-level functionality. The fundamental objective of tDCS is to alter neuron excitability by increasing or decreasing neural transmembrane voltage [15, 16]; without this level of biological abstraction included in a tDCS model, it is not possible to examine tDCS effects on brain cells within a computational simulation.

Beyond the tDCS modeling and simulation field, the computational neuroscience community possesses a large collection of biologically inspired, mathematical models of neural-level dynamics. The Hodgkin-Huxley model, for example, emulates voltage-gated ion channel functionality [17]. The Hindmarsh-Rose model incorporates neuron bursting, which is the diverse and chaotic behavior of rapid action potential spiking, believed to be very important in information encoding and propagation [18]. More recently, models have included individual neurotransmitter species, receptors, and binding kinetics to emulate neuron-neuron influences and communication [19].

A multiscale model that couples tDCS and cellular level functionality would enable researchers to simulate the impact that tDCS has on neurons. For instance, correlations between tDCS and ion channel functionality, action potential behavior, and neurotransmitter dynamics could be studied. In addition, patient-specific electrode configurations and treatment parameters could be optimized based on neuron behavior. Furthermore, a multiscale model would provide a bridge between the tDCS numerical simulation field and the computational neuroscience field, thereby enabling tDCS simulations access to the sophisticated, physiologically based cellular and subcellular models of the neuroscience community.

Several researchers have investigated the influence of extracellular electrical fields on the transmembrane voltage of individual and small groups of cells [15, 16, 20, 21]. At the organ level, Szmurło et al. [22, 23] demonstrated the applicability of the bidomain model [24] (see Section 2.1) to electroencephalograph (EEG) applications. They showed that this model, which has historically been used in cardiac applications, can reproduce scalp surface electric potential measurements originating from neuron action potentials.

In this paper, we combine these modeling strategies to produce a multiscale model of tDCS. We begin by coupling the bidomain model partial differential equations (PDEs) with boundary conditions that model tDCS treatments. We validate the model against several test cases on two different geometries. First, we simulate the model on an idealized two-dimensional domain, which provides a basic environment for visualizing and investigating electric potential and electric field characteristics. Then, we utilize an MRI-derived, three-dimensional human head geometry that possesses inhomogeneous and anisotropic tissue conductivities. In this setting, we examine the electric potential, electric current, electric field, and transmembrane voltage results produced from real-world tDCS electrode configurations. In both geometries, five distinct tissue types are used: skin, skull, cerebrospinal fluid (CSF), and the grey matter (GM) and white matter (WM) portions of the brain. Further, we detail the numerical methods and solution techniques that we implemented to enable reasonable simulation execution times.

To our knowledge, this paper presents the first multiscale tDCS model and simulations. We hope that the modeling and computational approaches presented in this paper help to expand tDCS simulation capabilities and further our understanding of tDCS impacts at the cellular level.

## 2. Materials and Methods

This section presents details of the model, numerics, and computational simulations used in this paper. First, an overview of the bidomain model is provided, as well as a description of the adaptation of this model for tDCS. Then, the numerical methods used to implement the multiscale tDCS model are described. Next, an overview of computational tools that we utilized is presented. Finally, the numerical experiments that were performed are described.

### 2.1. Bidomain Model

Modeling each cell in the brain and head is not computationally feasible; the bidomain model is based on a volume averaging approach, where the value at a point in a tissue is treated as an average over a minuscule, multicellular region around the point [25]. The bidomain model, as its name implies, models two domains, namely, the intracellular and extracellular spaces. Each of these domains is considered continuous within the brain, and they are insulated from each other by the cell membrane. Transcellular electric current is possible via ion channels in the cell membrane, and the transmembrane electric potential is defined to be the difference between intracellular and extracellular electric potentials, *v* = Φ_*i*_ − Φ_*e*_.

The bidomain model is given by the following system of partial differential equations for points in the brain, $\vec{x}\in \Omega_{B}$:

(2) $$\begin{array}{c} \nabla \cdot \left(M_{i}\nabla v\right)+\nabla \cdot \left(M_{i}\nabla \Phi_{e}\right)=\chi C_{m}\frac{\partial v}{\partial t}+\chi I_{\text{ion}}, \end{array}$$

(3) $$\begin{array}{c} \nabla \cdot \left(M_{i}\nabla v\right)+\nabla \cdot \left(\left(M_{i}+M_{e}\right)\nabla \Phi_{e}\right)=0, \end{array}$$

(4) $$\begin{array}{c} \frac{\partial \vec{s}}{\partial t}=F\left(\vec{s},v,t\right), \end{array}$$

where $\Phi_{e}=\Phi_{e}(\vec{x},t)$ is the extracellular electric potential and $v=v(\vec{x},t)$ is the transmembrane voltage. Note that, in this formulation, Φ_*i*_ has been eliminated with the substitution of *v*; if desired, Φ_*i*_ can be computed at any point in the domain using Φ_*i*_ = *v* + Φ_*e*_. **M**_*i*_ and **M**_*e*_ represent the intracellular and extracellular tissue conductivity tensor fields, respectively. In addition, *χ* is the cell membrane surface to volume ratio and *C*_*m*_ is the cell membrane capacitance. $I_{\text{ion}}=I_{\text{ion}}(\vec{s},v,t)$ is the total ionic current between the intracellular and extracellular domains, across the cell membrane. Equation (4), which characterizes the electrophysiological state of the neurons, can be represented by a single equation or by a system of ordinary differential equations (ODEs) [25].

Equations (2)–(4) are defined in the brain. Outside of the brain tissue, the scalp, skull, and cerebrospinal fluid are modeled as a passive conductor with the Laplace equation (1). In this extracerebral domain, neurons and other electrically responsive cells are not present, and so only the extracellular domain exists. Thus, the intracellular current is confined to the brain; this condition is enforced by requiring that the outflow of intracellular current from the brain into the extracerebral region equals zero:

(5) $$\begin{array}{c} \vec{n}\cdot \left(M_{i}\nabla \left(v+\Phi_{e}\right)\right)=0,\;\vec{x}\in \partial \Omega_{B}, \end{array}$$

where ∂*Ω*_*B*_ is the surface boundary of the brain.

Extracellular electric field continuity at the interface between the brain and extracerebral domain is preserved by requiring that $\vec{n}\cdot (M_{e}\nabla \Phi_{e})$ and Φ_*e*_ are continuous over ∂*Ω*_*B*_. To simplify notation, for the remainder of this paper Φ_*e*_ will be represented simply as Φ.

### 2.2. tDCS Adaptation

To make the bidomain model suitable for tDCS applications, two specific areas need to be addressed. First, the boundary conditions on the scalp must model tDCS administration. Second, cellular models that emulate neuron electrodynamics are necessary. The result of this adaptation is our multiscale tDCS model.

#### 2.2.1. Boundary Conditions

On the surface of the head, there are three separate boundary conditions needed to model tDCS. First, current delivered via tDCS anode electrodes is implemented by the nonhomogeneous Neumann boundary condition

(6) $$\begin{array}{c} \vec{n}\cdot M\nabla \Phi =I\left(\vec{x}\right), \end{array}$$

where $I(\vec{x})$ is the inward current at points on the boundary positioned under the anode electrode(s). Second, the cathode electrodes are given by the homogeneous Dirichlet condition

(7) $$\begin{array}{c} \Phi \left(\vec{x}\right)=0, \end{array}$$

for points on the boundary covered by the cathode electrode(s). All other points on the scalp surface are presumed insulated, and so the outward normal component of the current at these points must equal zero:

(8) $$\begin{array}{c} \vec{n}\cdot M\nabla \Phi =0. \end{array}$$

#### 2.2.2. Cell Model

**Figure 1:**
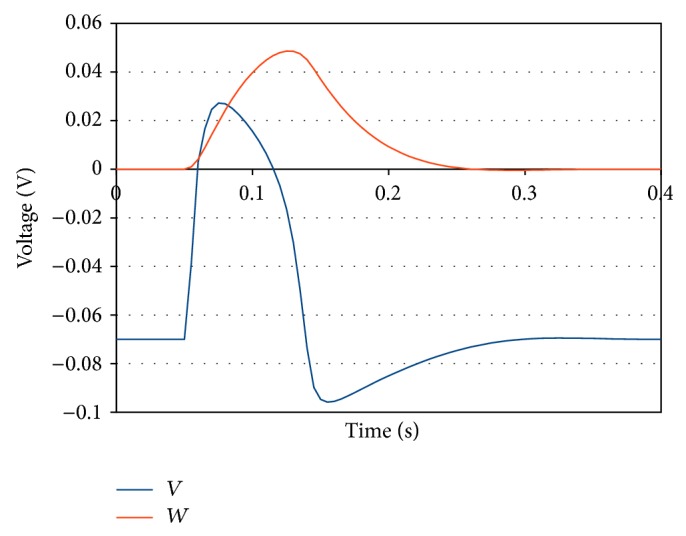
FitzHugh-Nagumo model action potential response.

Simulating single neuron transmembrane voltage dynamics was accomplished with the FitzHugh-Nagumo (FHN) model [26]:

(9a) ∂v∂t=c1vamp2(v−vrest)(v−vth)(vpeak−v)−(c2w)+Iapp,

(9b) ∂w∂t=b(v−vrest−c3w),

where *v* is again the transmembrane voltage and *w* is a state variable that controls transmembrane voltage repolarization. Here, the threshold voltage is defined as *v*_th_ = *v*_rest_  +  *av*_amp_, and *v*_amp_ is the difference between peak and resting membrane voltages, *v*_amp_ = *v*_peak_ − *v*_rest_. We used *a* = 0.13, *b* = 13.0, *c*_1_ = 260.0, *c*_2_ = 100.0, and *c*_3_ = 1.0, as proposed by FitzHugh [26] with the coefficients scaled for seconds. This implementation of the FHN model allows us to define *v*_rest_ and *v*_peak_, which we set to −0.07 V and 0.04 V, respectively.  Figure 1 displays an AP response of the FHN model when given an applied current *I*_app_ for *t* ∈ [0.05,0.06].

The PDE system (2)–(4) and this cell model couple through the the right-hand side of (2) and (9a). Also, when using the FHN model with the bidomain model, (4) is represented by the single state equation (9b).

### 2.3. Numerical Implementation

The multiscale tDCS model was solved with a Godunov operator splitting scheme [25]. The solution algorithm consists of the following two steps.

(1) Solve the ODE system:
  (10) ∂v∂t=c1vamp2(v−vrest)(v−vth)(vpeak−v)−(c2w),∂w∂t=b(v−vrest−c3w),
  for *t*_*n*_ < *t* ≤ *t*_*n*_ + Δ*t*, and *v*(*t*_*n*_) and *w*(*t*_*n*_) are known. Let ${\tilde{v}}_{n}$ denote the partial solution of *v* at step *t*_*n*_ + Δ*t*.
(2) Solve the PDE system:
  (11) ∇·(MiχCm∇v)+∇·(MiχCm∇Φ)=∂v∂t, x→∈ΩB,∇·(MiχCm∇v)+∇·[(MiχCm+MeχCm)∇Φ]=0,x→∈ΩB,∇·(MeχCm∇Φ)=0, x→∈Ω∖ΩB,
  for *t*_*n*_ < *t* ≤ *t*_*n*_ + Δ*t*, $v(t_{n})={\tilde{v}}_{n}$, and boundary conditions are specified in Sections 2.1 and 2.2.

The result is numerical solutions of *v* and Φ at time step *t*_*n*+1_ = *t*_*n*_ + Δ*t*. This fractional step method decouples the nonlinear ODEs from the PDEs. This is advantageous since the ODE system can then be evaluated more frequently than the PDEs during periods of rapid transmembrane voltage change, that is, AP spiking, without having to also solve the computationally intensive PDE system. In addition, this Godunov splitting scheme is numerically stable and computationally efficient [27].

The ODE system in step (1) is solved with Heun's method [28]. The PDE system in step (2) is solved as a coupled system, discretizing in time with the implicit Euler method, and in space with the finite element method [29]. The resulting finite element formulation yields the following system of equations in block matrix form [25]:

(12) $$\begin{array}{c} \left(\begin{array}{cc} A & B\\ B^{T} & C \end{array}\right)\left(\begin{array}{c} \vec{v}\\ \vec{\Phi } \end{array}\right)=\left(\begin{array}{c} \vec{\alpha }\\ \vec{0} \end{array}\right), \end{array}$$

where the *i*th and *j*th entries are given by

(13) Aij=∫ΩBNjNidx+ΔtχCm∫ΩBMi∇Nj·∇Nidx,Bij=ΔtχCm∫ΩBMi∇Nj·∇Nidx,Cij=ΔtχCm∫ΩB(Mi+Me)∇Nj·∇Nidx+ΔtχCm∫Ω∖ΩBMe∇Nj·∇Nidx,αi=∫ΩBvnNidx,

and *v*_*j*_ and Φ_*j*_ are the unknown transmembrane and electric potentials that together form the solution that we seek. Here, *N*_*i*_ and *N*_*j*_ are finite element basis functions over the discretized domain.

The linear system (12) is solved by the conjugate gradient method [30] preconditioned with the following block preconditioner [29]:

(14) $$\begin{array}{c} K=\left(\begin{array}{cc} \tilde{A^{-1}} & 0\\ 0 & \tilde{C^{-1}} \end{array}\right), \end{array}$$

where $\tilde{A^{-1}}$ and $\tilde{C^{-1}}$ are incomplete LU (ILU) factorizations [31] of **A** and **C**, respectively.

### 2.4. Computational Tools

**Figure 2:**
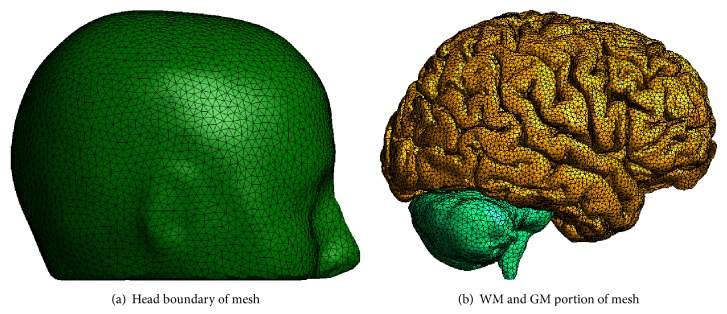
Portions of the computational grid used in multiscale tDCS simulations.

Several of the multiscale tDCS numerical simulations are performed on a three-dimensional grid derived from human MRI data. The SimNIBS software package [32] provides the associated computational grid; it is a high-quality tetrahedral mesh of the scalp, skull, CSF, GM, and WM. Gmsh [33] supported mesh visualization and supplied grid file conversion to a format supported by Diffpack (http://www.diffpack.com/) [34].  Figure 2 displays portions of the computational mesh used in the three-dimensional simulations.

Finite element solutions were performed with Diffpack. An anisotropic conductivity tensor field for the brain region of the MRI-derived mesh is generated by SimNIBS and stored in a Matlab [35] binary data file; the Matlab engine was invoked and utilized in our C++ code to access these tensor data at run time. The ODE solver (Heun's method) was implemented in C++, and the operator splitting scheme was performed with Diffpack. In addition, the block coefficient matrix (12) and block preconditioner (14) were implemented with Diffpack. Results were exported to VTK format and visualized with ParaView (http://www.paraview.org/).

### 2.5. Multiscale tDCS Numerical Experiments

Numerical experiments were contrived to examine the following four properties:

1. action potential conduction velocity;
2. tDCS electric potential;
3. tDCS electric current and field;
4. tDCS-induced transmembrane voltage increase.

Experiments were run with a global time-step Δ*t* = 1 ms. The ODE system was solved more frequently with a time step Δ*t*_ODE_ = 0.5 ms. At each Δ*t* time step, the system of (12) was solved using the conjugate gradient method preconditioned with a relaxed ILU block matrix (14), with relaxation parameter *ω* = 0.5 [31] for both the $\tilde{A^{-1}}$ and $\tilde{C^{-1}}$ blocks. A relative residual convergence monitor of ||*r* ^*k*^||/||*r* ^0^|| was used, where ||*r* ^0^|| is the norm of the initial residual, and ||*r* ^*k*^|| is the norm of the residual of the *k*th iteration. The convergence tolerance was set to 10^−8^. In all experiments, the parameter *C*_*m*_ was set to 1 × 10^−4^ (F/m^2^), and *χ* was set to 1.26 × 10^5^ (1/m) [36].

The multiscale tDCS model was assessed and validated against several two- and three-dimensional numerical experiments. The following subsections describe each of these.

#### 2.5.1. Two-Dimensional Experiments

**Figure 3:**
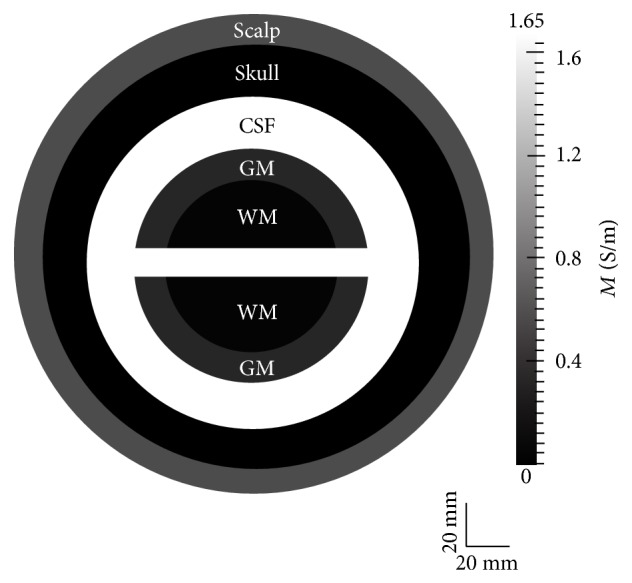
Two-dimensional geometry used in multiscale tDCS simulations; the gray scale illustrates the electrical conductivity of the different tissue types.

Figure 3 displays the geometry used for two-dimensional experiments. It is constructed with concentric annuli to simulate the overlapping and embedded nature of head and cerebral tissues. The innermost region, emulating the white matter of the brain, is a circle with radius = 40 mm. Surrounding this region, four annuli are positioned with outer radii equal to 50, 70, 90, and 100 mm, which emulate the GM, CSF, skull, and scalp tissues, respectively. To simulate the interwoven nature of the CSF with the GM and WM, a 10 mm thick CSF strip extends horizontally through the center of the geometry, providing a passage for CSF through the GM and WM. This prototypical domain allows us to observe and comprehensively assess action potential conduction, electric potential, and electric field simulation results throughout the entire domain.

Isotropic extracellular conductivities were assigned to different tissues: skin = 0.465, skull = 0.010, CSF = 1.654, GM = 0.276, and WM = 0.126, each with units S/m [37], and an intracellular conductivity value of 0.1 S/m was used in the GM and WM. Each experiment included 10,000 linear triangular finite elements.

Individual two-dimensional experiments are described in the following paragraphs.

 Action potential conduction: an AP centered at (0, −25), encompassing a circular region with radius = 2.5 mm, was simulated for *t* ∈ (0,10] ms. Total simulation time is 100 ms. tDCS was not administered and so the homogeneous Neumann boundary condition $\vec{n}\cdot M_{e}\nabla \Phi =0$ was imposed on the entire scalp surface. This experiment is used to ensure the biological legitimacy of the multiscale model's AP conduction velocity and in doing so verifies that appropriate parameter values were selected. This experiment is also used to examine the electric potential, Φ, produced by the AP.
 tDCS electric potential and field: models of tDCS based on the Laplace equation (1) accurately quantify tDCS electric potentials and fields [11, 14]. Therefore, to validate the multiscale model, its electric potential and electric field simulation results are compared to those produced by (1).
 These comparisons are performed using two simulations. First, tDCS was simulated with the anode and cathode electrodes positioned at (−100,0) and (70.7,70.7), respectively. Electrode size is 10 mm, and the anode electric current magnitude was set to 1.0 mA (see Section 2.2.1) at *t* = 0. No AP was artificially initiated; that is, *I*_app_ = 0 (9a), and simulation duration is 100 ms.
 This numerical experiment was repeated with a different electrode configuration, placing the anode electrode at (−70.7,70.7) and the cathode at (0, −100). This electrode arrangement was selected to provide substantive differences from the first arrangement. First, the electric current entry via the anode does not neighbor the central CSF channel, as is the case with the first electrode configuration. Second, the current exit at the cathode electrode is as distant from the central CSF channel as possible in this two-dimensional domain. In addition, the anode and cathode are on opposite sides of the CSF channel in the second configuration.

#### 2.5.2. Three-Dimensional Experiments

Three-dimensional experiments were conducted on an MRI-derived volume mesh.  Figure 2 displays portions of the mesh used in these simulations. Extracellular conductivities of the scalp, skull, and CSF were isotropic and set to 0.465 S/m, 0.010 S/m, and 1.654 S/m, respectively [37]. Anisotropic extracellular conductivities of the GM and WM portions of the brain were used; this tensor field is provided by the SimNIBS software package (see Section 2.4). The intracellular conductivity for the brain region was set to 0.1 S/m.

Three separate electrode montages were selected for the three-dimensional simulations (see Table 1). Each montage is specified using the international 10-20 system [38]. Transcranial direct current stimulation applied with montage 1 has shown to enhance motor sequence learning [39], for example. This montage is known to target the motor cortex region ipsilateral to the anode electrode [40]. Montage 2 has been utilized in a host of biomedical research studies involving motor skills and also enhances neural tissue excitability in the motor cortex ipsilateral to the anode [38]. Montage 3 has been shown to improve gait and bradykinesia in patients with Parkinson's disease [3]. However, the regions of the brain and the mechanisms by which tDCS enhances motor performance in these individuals remain unclear. Neurostimulation research suggests that stimulation of the primary motor cortex is a catalyst for motor-skill improvement [3, 41]. In addition, other research studies verify that electrical stimulation to the subthalamic nucleus (STN) and substantia nigra (SN) greatly improves motor performance in Parkinson's disease patients [42–46].

In all montages, the anode electric current magnitude was set to 1.0 mA (see Section 2.2.1) at *t* = 0 and the surface area of each electrode is approximately 25 cm^2^ [3, 39]. Numerical experiments were run for 100 ms, and no APs were forced; that is, *I*_app_ = 0 (9a). The head geometry is comprised of approximately 1.1 million linear tetrahedra finite elements.

Again, the Laplace equation (1) accurately models tDCS electric potentials and fields [11, 14]. For each montage, the multiscale model is validated by comparing its scalp surface electric potential simulation results against those generated by Laplace equation-based simulations. A similar comparison is performed with the tDCS electric current and field. Then, the multiscale model's ability to predict the areas of the brain that become more excitable from tDCS treatments administered with these three montages is verified. This is accomplished by examining the transmembrane voltage increase in those regions of the brain known to become more excitable from tDCS. For montages 1 and 2, the motor cortex ipsilateral to the anode is examined, and for montage 3 the motor cortex and the STN and SN regions are inspected (see Table 1).

## 3. Results and Discussion

### 3.1. Two-Dimensional Simulations

#### 3.1.1. Action Potential Conduction

**Figure 4:**
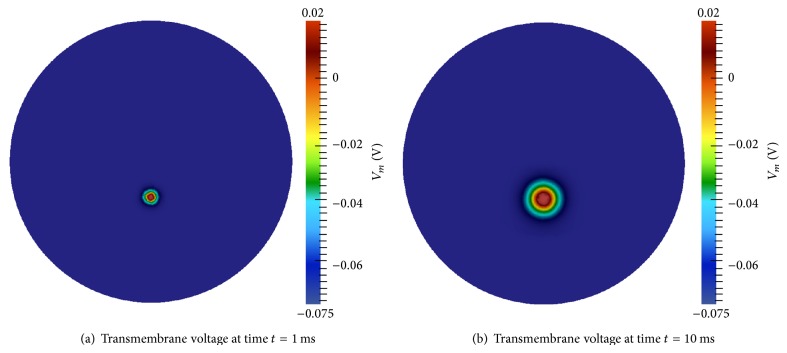
Action potential conduction in two-dimensional geometry.

Transmembrane voltage results for the AP numerical experiment described in Section 2.5.1 are presented in Figure 4.  Figure 4(a) shows the start of the AP. By time *t* = 10 ms, AP dispersion is quite noticeable (Figure 4(b)). The conduction velocity is approximately 2.0 m/s. This value is on the lower end of normal neural conduction velocities [47]; however, average AP speed varies among individuals and testing conditions [48]. In addition, the conduction velocity can easily be adjusted in the model by changing *χ*, *C*_*m*_, **M**_*i*_, or **M**_*e*_. Further, alternative neuron models possess different AP transmembrane voltage upstroke rates that will affect conduction velocity [25].

**Figure 5:**
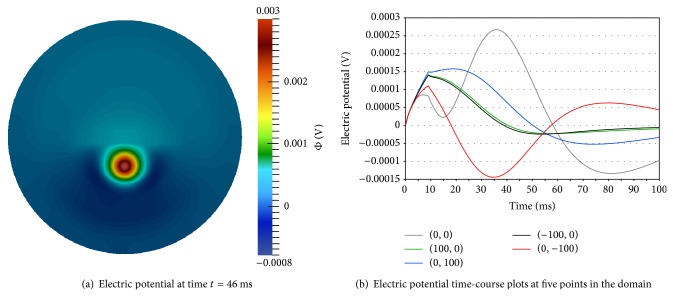
Electric potential (Φ) from action potential.

Figure 5 displays the electric potential, Φ, produced from the AP.  Figure 5(a) shows Φ throughout the entire two-dimensional domain at *t* = 46 ms. Variability in the electric potential due to the inhomogeneous extracellular conductivities is noticeable.  Figure 5(b) displays electric potential time-course plots, for points at the center of the domain and surface boundary that intersect each Cartesian axis. The curves representing the points that intersect the *x*-axis, namely, (−100,0) and (100,0), are similar due to their symmetry with respect to the AP. However, all other curves show variability due to their spacial separation and tissue conductivity inhomogeneity.

These electric potential results are of the same order of magnitude as those reported by Szmurło et al. [22]. Further, they are several orders of magnitude lower than those produced during tDCS sessions [37]. These results are consistent with the observation that head surface electric potential measurements are dominated by the tDCS electric current with negligible impact from AP conduction [49].

This numerical experiment confirms that the selected parameter set produces biologically reasonable action potential results. Conduction speeds are appropriate and the electric potential resulting from an AP is consistent with previous research reports. In the following sections, the multiscale model is validated when tDCS is administered.

#### 3.1.2. tDCS Administration

**Figure 6:**
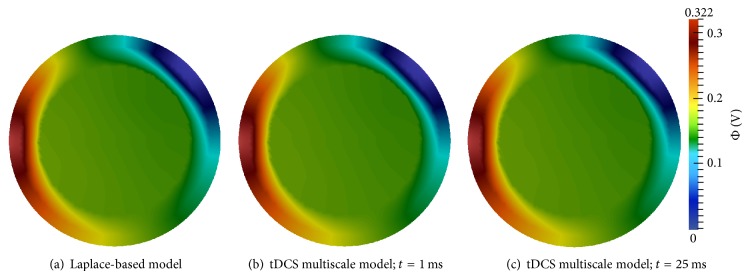
Electric potential (Φ) results for the first tDCS electrode configuration; anode at (−100,0) and cathode at (70.7,70.7).

*First Electrode Configuration*. Electric potential simulation results for the Laplace equation-based model and the multiscale model are presented in Figure 6. The electric potential of the Laplace model (Figure 6(a)) closely resembles the multiscale model's electric potential at both *t* = 1 ms (Figure 6(b)) and *t* = 25 ms (Figure 6(c)). The electric potential difference between these two times has minuscule change. For *t* > 25 ms, the electric potential stabilizes, and no visible differences were observed throughout the remainder of the simulation.

**Figure 7:**
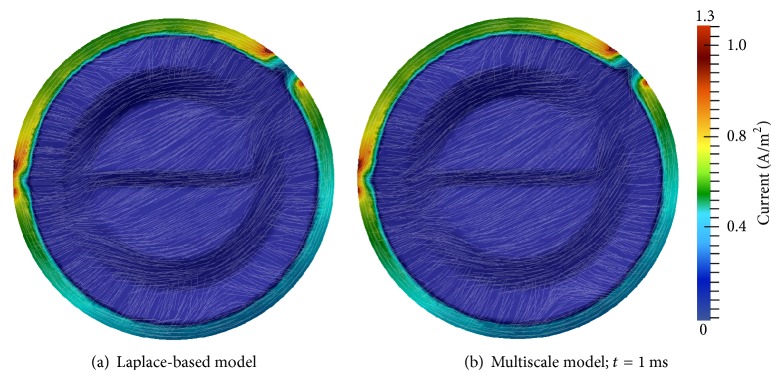
Electric field results for the first tDCS electrode configuration: anode at (−100,0) and cathode at (70.7,70.7); the color bar specifies current density and streamlines show electric field direction.

Figure 7 displays the tDCS electric fields for the Laplace and multiscale models. Multiscale model results are shown at *t* = 1 ms, but they are essentially identical at all times. Differences between the models' electric current densities and fields are virtually indistinguishable. The tendency of the electric field to shunt the skull is due to the low conductivity of this tissue, and this produces an increased current density exiting the edges of the anode and entering the edges of the cathode [11]. In addition, the portion of the electric current that penetrates the skull has high affinity for the highly conductive CSF.

**Figure 8:**
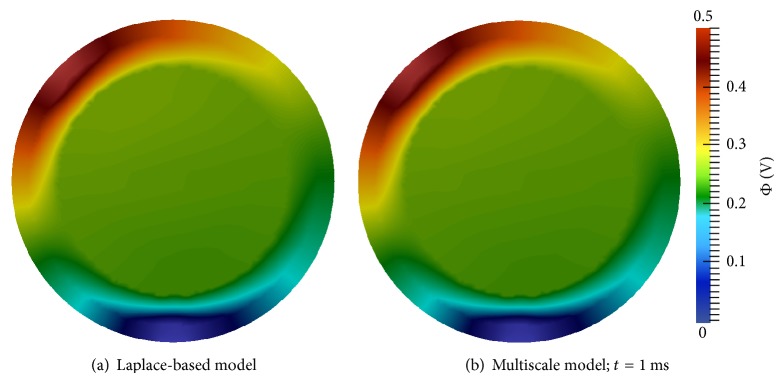
Electric potential (Φ) results for the second tDCS electrode configuration: anode at (−70.7,70.7) and cathode at (0, −100).

**Figure 9:**
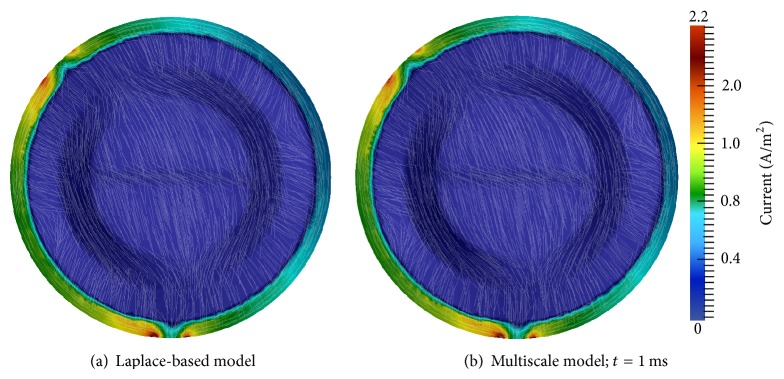
Electric field results for the second tDCS electrode configuration: anode at (−70.7,70.7) and cathode at (0, −100); the color bar specifies current density and streamlines show electric field direction.

*Second Electrode Configuration*. Figures 8 and 9 display electric potential and electric field results for both models with tDCS delivered with the second electrode configuration. The multiscale simulation results again match the Laplace-based simulation results very closely. Electric field shunting is again present as well as the resulting areas of higher current density at the borders of the electrodes. Perhaps more visible in this electrode configuration is the propensity of the current to gravitate towards CSF regions of the domain (Figure 9). Similar to the first configuration, electric potential, current, and field results of the multiscale model were essentially identical at all time steps.

These two experiments demonstrate that the multiscale tDCS model can accurately compute electric potentials and fields when tDCS is administered. In the next section these validations are continued. In addition, the ability of the multiscale model to accurately identify regions of the brain that are electrically excited by tDCS is also demonstrated.

### 3.2. Three-Dimensional Simulations

#### 3.2.1. Montage 1

**Figure 10:**
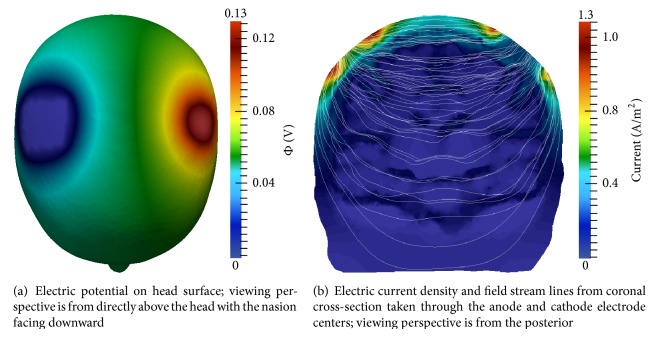
Multiscale model electric potential and current simulation results using montage 1; *t* = 1 ms.

Figure 10 displays the electric potential and field results of the multiscale model simulated with the montage 1 electrode configuration (see Table 1). Maximum and minimum surface potential coincide with electrode location (Figure 10(a)).  Figure 10(b) illustrates the electric current and field within a coronal cross-section through the C3 and C4 electrodes, viewed from the posterior. Curvilinear electric field lines within the cerebral tissue due to the interwoven CSF and GM and WM tissues are visible.

The shunting of the electric field along the scalp and skull is noticeable in Figure 10(b), resulting in regions of higher current density at the electrode edges, similar to the two-dimensional simulation results (see Section 3.1). Further, electric potential, current, and field results are essentially the same at all time steps, as was observed in the two-dimensional experiments. These results are in agreement with simulation results produced by Laplace equation-based models.

**Figure 11:**
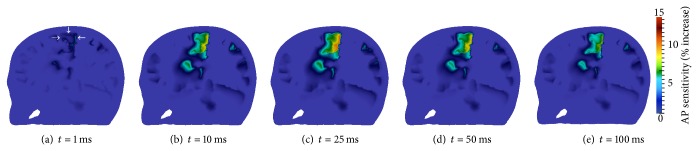
Transmembrane voltage increase in sagittal cross-section through motor cortex ipsilateral to the anode; viewing perspective is from the left side with head facing left. The arrows in (a) locate the primary motor cortex.

Figure 11 displays transmembrane voltage results for this montage. A sagittal cross-section through the motor cortex ipsilateral to the anode electrode and perpendicular to the primary electric field direction was taken. Viewing perspective is from the left side of the head with the head facing left. The arrows (Figure 11(a)) locate the motor cortex, which is the area of the brain expected to have increased excitability from tDCS (see Table 1). Results are displayed for *t* = 1, 10, 25, 50, and 100 ms. The increased sensitivity of neural tissue to generate action potentials was quantified as a percentage with the following formula:

(15) $$\begin{array}{c} \text{AP}\;\;\text{sensitivity}=\frac{v_{\text{rest}}-v}{v_{\text{rest}}-v_{\text{th}}}\times 100\%, \end{array}$$

where *v*_rest_ = −70 mV and *v*_th_ = −55.7 mV, given the parameters used with the FHN cell model (see Section 2.2.2). This formula provides a measure of the degree to which neural tissue has increased from its resting membrane potential to become more susceptible to firing action potentials.

After 1 ms of tDCS administration (Figure 11(a)), increases in resting potential are noticed throughout the cerebral tissue, most notably in the motor cortex. At time *t* = 10 ms (Figure 11(b)), AP sensitivity in portions of the motor cortex has increased by approximately 8%. By 25 ms (Figure 11(c)), the effects of tDCS are quite visible. Again the greatest increase in sensitivity is achieved in the motor cortex, with the majority of this region having values over 5% and portions exceeding 10%. After 25 ms, the membrane potential begins to repolarize (Figure 11(d)). This process is slow, and by the end of the simulation (Figure 11(e)), resting membrane potential is still elevated in the motor cortex.

#### 3.2.2. Montage 2

**Figure 12:**
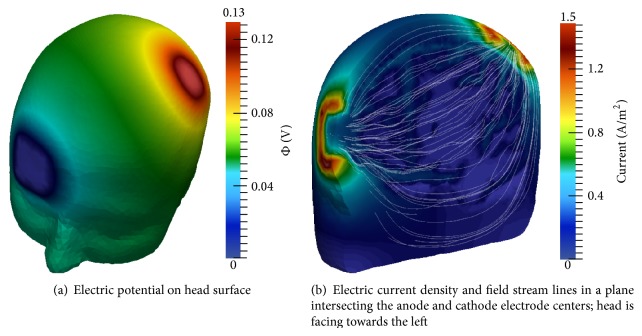
Multiscale model electric potential and current simulation results using montage 2; *t* = 1 ms.

Montage 2 electric potential and electric field results are presented in Figure 12. Maximum and minimum surface potential again coincide with anode and cathode electrode placement, respectively (Figure 12(a)). The current density and direction are viewed from a plane intersecting the anode and cathode centers (Figure 12(b)). The electric field shunts along the skull, as was observed in montage 1, again resulting in higher current magnitudes at the borders of the electrodes. Wave-like electric field lines through the interwoven CSF, GM, and WM are also visible. These results are in agreement with those generated by Laplace equation-based models.

**Figure 13:**
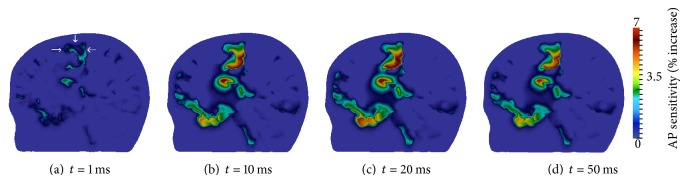
Transmembrane voltage increase in plane longitudinally through the motor cortex ipsilateral to the anode; viewing perspective is from the left posterior with the head facing towards the left. The arrows in (a) locate the primary motor cortex.

Figure 13 displays the transmembrane voltage results for montage 2. A slice longitudinal through the motor cortex ipsilateral to the anode, approximately perpendicular to the primary electric field path, was taken. Viewing perspective is from the left posterior of the head, with the head facing left. The arrows (Figure 13(a)) locate the motor cortex ipsilateral to the anode, the expected region of increased action potential sensitivity. Results are displayed for *t* = 1, 10, 20, and 50 ms.

The multiscale simulation predicts an increase in transmembrane voltage in the motor cortex after 1 ms of tDCS treatment (Figure 13(a)), and AP sensitivity increases near 7% are visible at 10 ms (Figure 13(b)). The maximum increase in resting membrane voltage for this montage occurs at 20 ms (Figure 13(c)). Repolarization occurs for *t* > 20 ms and is quite observable at 50 ms ( Figure 13(d)).

Montages 1 and 2 possess similar transmembrane voltage trends in the motor cortex region. The simulations predict that montage 1 will, however, increase the resting membrane voltage in this region approximately 1.5 times that of montage 2. This phenomena can be explained by the fact that the electric current distribution with montage 1 is more confined to this locality due to the closer proximity of its electrodes to each other and to the motor cortex [11]. Supporting this explanation is the observation that tDCS medical research studies fundamentally use montage 1 with motor cortex specific applications, whereas montage 2 is also utilized in other treatment focuses [38].

#### 3.2.3. Montage 3

**Figure 14:**
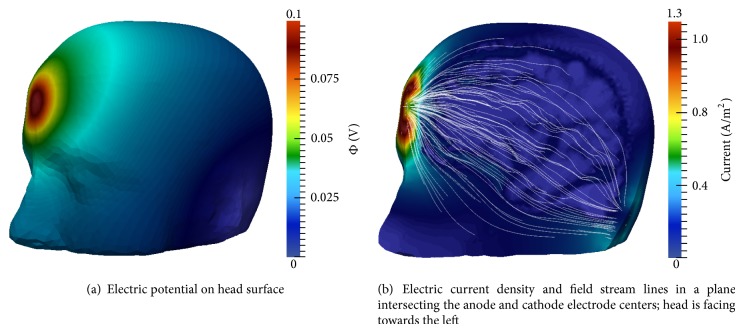
Multiscale model electric potential and current simulation results using montage 3; *t* = 1 ms.

Figure 14 displays surface potential and electric field simulation results for the third montage. The patient's left mastoid is shown; an identical cathode is positioned over the contralateral mastoid. The electric current and field are displayed in a cross-section through the centers of the anode and left mastoid cathode. Once again, the current reaches maximal values at electrode edges, and both skull-divergent and convoluted cerebral electric field lines are present. These results are consistent with those generated by Laplace-based models.

**Figure 15:**
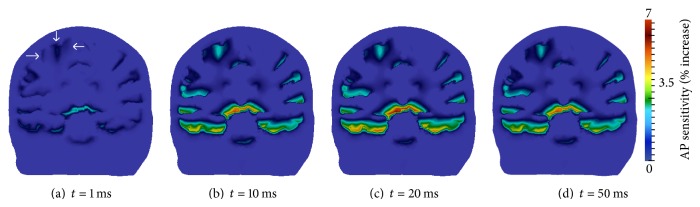
Transmembrane voltage increase in plane through left motor cortex viewed from the back of the head. The arrows in (a) locate the primary motor cortex region.

Based on the research communities' suggestion that motor cortex stimulation enhances mobility and movement capabilities in Parkinson's disease patients (see Section 2.5.2), we first examined the increase in transmembrane voltage in this region (Figure 15). A plane through the left primary motor cortex was taken; viewing perspective is from the rear. The motor cortex is indicated by the arrows (Figure 15(a)), and results are displayed for *t* = 1, 10, 20, and 50 ms.

Increases in motor cortex excitability are observable at 10 ms (Figure 15(b)) and reach maximal values at 20 ms (Figure 15(c)). Repolarization begins after this time and is noticeable at 50 ms (Figure 15(d)). Although an increase in the resting membrane potential of the motor cortex is visible throughout this simulation, the increase is low when compared to the previous two montages. Specifically, AP sensitivity increases do not exceed 2.0%, which is less than 50% attained by montage 2 and less than 25% attained by montage 1.

**Figure 16:**
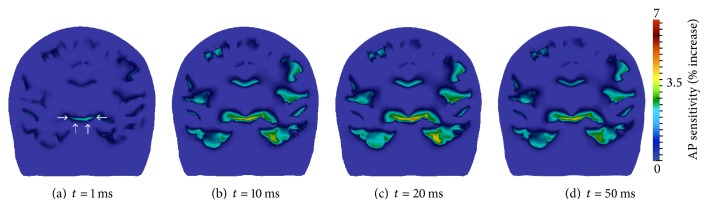
Transmembrane voltage increase in coronal slice through the STN and SN, viewed from the back of the head. The arrows in (a) locate the STN and SN regions.

Next, the increase in membrane resting potential in the subthalamic nucleus and substantia nigra regions (Figure 16) were examined, due to results from deep brain stimulation research affirming that electrically stimulating these regions yields enhanced motor abilities (see Section 2.5.2). A coronal slice through the STN and SN regions is shown, viewed from the posterior. Results are again displayed for *t* = 1, 10, 20, and 50 ms.

Resting membrane voltage increases in these regions are much larger than those seen in the motor cortex, with AP sensitivity values comparable to those attained with montages 1 and 2. After 1 ms of tDCS administration, AP sensitivity increases in the STN and SN regions are observable (Figure 16(a)). By 10 ms (Figure 16(b)), AP sensitivity in these regions is approaching 4 percent. Maximal increases occur once again at 20 ms (Figure 16(c)), and after 20 ms membrane voltage begins to repolarize (Figure 16(d)).

These three-dimensional numerical experiments further validate the multiscale model's ability to accurately compute the electric potentials and currents generated during tDCS treatments. In addition, using an MRI-derived head geometry and anisotropic tissue conductivities, the ability of the multiscale model to identify regions in the brain that have elevated resting membrane potentials during tDCS treatments with three real-world electrode configurations has been shown.

## 4. Conclusions

We have presented a novel, multiscale model of tDCS that couples the mathematics of this procedure to neuronal functioning. The model has been validated against several test cases with comparisons to existing simulations and medical research results. In all of these experiments, the multiscale model accurately simulates tDCS electric potentials and electric fields. We verified the model's ability to correctly identify those areas of the brain known to be electrically stimulated by specific, real-world tDCS electrode montages. Further, we demonstrated the model's medical applicability with simulations on a three-dimensional head geometry, derived from MRI data, with anisotropic and inhomogeneous tissue conductivities.

To our knowledge, this paper presents the first multiscale model and simulations of tDCS, which effectively couples cellular-level functionality with tDCS treatment conditions. In addition, our simulation implementation strategies provide an intersection between the tDCS simulation and computational neuroscience communities. In the future, we plan to enhance the fidelity of our simulations with more robust, location-specific neuron models. We also plan to investigate alternative electrode configurations and the numerical methods that most efficiently execute these simulations.

## Figures and Tables

**Table 1:** Multiscale tDCS three-dimensional numerical experiment electrode configurations, specified using the international 10-20 system.

	Anode	Cathode(s)	Target region
Montage 1	C3	C4	Motor cortex (ipsilateral to anode)
Montage 2	C3	Fp2	Motor cortex (ipsilateral to anode)
Montage 3	Forehead symmetric	Mastoids (both)	Motor cortex; STN and SN

## References

[1] Boggio P. S., Khoury L. P., Martins D. C. S., Martins O. E. M. S., de Macedo E. C., Fregni F. (2009). Temporal cortex direct current stimulation enhances performance on a visual recognition memory task in Alzheimer disease. *Journal of Neurology, Neurosurgery and Psychiatry*.

[2] Boggio P. S., Valasek C. A., Campanhã C., Giglio A. C. A., Baptista N. I., Lapenta O. M., Fregni F. (2011). Non-invasive brain stimulation to assess and modulate neuroplasticity in Alzheimer's disease. *Neuropsychological Rehabilitation*.

[3] Benninger D. H., Lomarev M., Lopez G., Wassermann E. M., Li X., Considine E., Hallett M. (2010). Transcranial direct current stimulation for the treatment of Parkinson's disease. *Journal of Neurology, Neurosurgery and Psychiatry*.

[4] Boggio P. S., Ferrucci R., Rigonatti S. P., Covre P., Nitsche M., Pascual-Leone A., Fregni F. (2006). Effects of transcranial direct current stimulation on working memory in patients with Parkinson's disease. *Journal of the Neurological Sciences*.

[5] Kalu U. G., Sexton C. E., Loo C. K., Ebmeier K. P. (2012). Transcranial direct current stimulation in the treatment of major depression: a meta-analysis. *Psychological Medicine*.

[6] Fregni F., Boggio P. S., Nitsche M. A., Rigonatti S. P., Pascual-Leone A. (2006). Cognitive effects of repeated sessions of transcranial direct current stimulation in patients with depression. *Depression and Anxiety*.

[7] Schlaug G., Renga V., Nair D. (2008). Transcranial direct current stimulation in stroke recovery. *Archives of Neurology*.

[8] Hesse S., Waldner A., Mehrholz J., Tomelleri C., Pohl M., Werner C. (2011). Combined transcranial direct current stimulation and robot-assisted arm training in subacute stroke patients: an exploratory, randomized multicenter trial. *Neurorehabilitation and Neural Repair*.

[9] Neuling T., Wagner S., Wolters C. H., Zaehle T., Herrmann C. S. (2012). Finite-element model predicts current density distribution for clinical applications of tDCS and tACS. *Frontiers in Psychiatry*.

[10] Gasca F., Marshall L., Binder S., Schlaefer A., Hofmann U. G., Schweikard A. Finite element simulation of transcranial current stimulation in realistic rat head model.

[11] Datta A., Zhou X., Su Y., Parra L. C., Bikson M. (2013). Validation of finite element model of transcranial electrical stimulation using scalp potentials: implications for clinical dose. *Journal of Neural Engineering*.

[12] Miranda P. C., Lomarev M., Hallett M. (2006). Modeling the current distribution during transcranial direct current stimulation. *Clinical Neurophysiology*.

[13] Kessler S. K., Minhas P., Woods A. J., Rosen A., Gorman C., Bikson M. (2013). Dosage considerations for transcranial direct c urrent stimulation in children: a computational modeling study. *PLoS ONE*.

[14] Plonsey R., Heppner D. B. (1967). Considerations of quasi-stationarity in electrophysiological systems. *The Bulletin of Mathematical Biophysics*.

[15] Sadleir R. (2010). A bidomain model for neural tissue. *International Journal of Bioelectromagnetism*.

[16] Mandonnet E., Pantz O. (2011). The role of electrode direction during axonal bipolar electrical stimulation: a bidomain computational model study. *Acta Neurochirurgica*.

[17] Hodgkin A. L., Huxley A. F. (1952). A quantitative description of membrane current and its application to conduction and excitation in nerve. *The Journal of Physiology*.

[18] Hindmarsh J. L., Rose R. M. (1984). A model of neuronal bursting using three coupled first order differential equations. *Proceedings of the Royal Society of London Series B: Biological sciences*.

[19] Koch C. (1998). *Methods in Neuronal Modeling: From Ions to Networks*.

[20] Ying W., Henriquez C. S. (2007). Hybrid finite element method for describing the electrical response of biological cells to applied fields. *IEEE Transactions on Biomedical Engineering*.

[21] Agudelo-Toro A., Neef A. (2013). Computationally efficient simulation of electrical activity at cell membranes interacting with self-generated and externally imposed electric fields. *Journal of Neural Engineering*.

[22] Szmurło R., Starzyński J., Sawicki B., Wincenciak S. Multiscale finite element model of the electrically active neural tissue.

[23] Szmurło R., Starzyński J., Sawicki B., Wincenciak S., Cichocki A. Bidomain formulation for modeling brain activity propagation.

[24] Geselowitz D. B., Miller W. T. (1983). A bidomain model for anisotropic cardiac muscle. *Annals of Biomedical Engineering*.

[25] Sundnes J., Lines G. T., Cai X., Nielsen B. F., Mardal K.-A., Tveito A. (2006). *Computing the Electrical Activity in the Heart*.

[26] FitzHugh R. (1961). Impulses and physiological states in theoretical models of nerve membrane. *Biophysical Journal*.

[27] Southern J. A., Plank G., Vigmond E. J., Whiteley J. P. (2009). Solving the coupled system improves computational efficiency of the bidomain equations.. *IEEE transactions on bio-medical engineering*.

[28] Suli E. (2003). *An Introduction to Numerical Analysis*.

[29] Mardal K. A., Sundes J., Langtangen H. P., Tveito A., Langtangen H. P., Tveito A. (2003). Systems of PDEs and block preconditioning. *Advanced Topics in Computational Partial Differential Equations: Numerical Methods and Diffpack Programming*.

[30] van der Vorst H. A. (2003). *Iterative Krylov Methods for Large Linear Systems*.

[31] Langtangen H. P. (2003). *Computational Partial Differential Equations: Numerical Methods and Diffpack Programming*.

[32] Windhoff M., Opitz A., Thielscher A. (2013). Electric field calculations in brain stimulation based on finite elements: An optimized processing pipeline for the generation and usage of accurate individual head models. *Human Brain Mapping*.

[33] Geuzaine C., Remacle J. F. (2009). Gmsh: A 3-D finite element mesh generator with built-in pre- and post-processing facilities. *International Journal for Numerical Methods in Engineering*.

[34] Bruaset A. M., Langtangen H. P. Diffpack: a software environment for rapid prototyping of PDE solvers.

[35] MATLAB (2013). *Version 8.2.0.701 (R2013b)*.

[36] Szmurlo R., Starzynski J., Stanislaw S., Rysz A. (2009). Numerical model of vagus nerve electrical stimulation. *COMPEL*.

[37] Datta A., Baker J. M., Bikson M., Fridriksson J. (2011). Individualized model predicts brain current flow during transcranial direct-current stimulation treatment in responsive stroke patient. *Brain Stimulation*.

[38] Nitsche M. A., Cohen L. G., Wassermann E. M., Priori A., Lang N., Antal A., Paulus W., Hummel F., Boggio P. S., Fregni F., Pascual-Leone A. (2008). Transcranial direct current stimulation: State of the art 2008. *Brain Stimulation*.

[39] Kang E. K., Paik N.-J. (2011). Effect of a tDCS electrode montage on implicit motor sequence learning in healthy subjects. *Experimental and Translational Stroke Medicine*.

[40] Okamoto M., Dan H., Sakamoto K., Takeo K., Shimizu K., Kohno S., Oda I., Isobe S., Suzuki T., Kohyama K., Dan I. (2004). Three-dimensional probabilistic anatomical cranio-cerebral correlation via the international 10–20 system oriented for transcranial functional brain mapping. *NeuroImage*.

[41] Fregni F., Boggio P. S., Santos M. C., Lima M., Vieira A. L., Rigonatti S. P., Silva M. T. A., Barbosa E. R., Nitsche M. A., Pascual-Leone A. (2006). Noninvasive cortical stimulation with transcranial direct current stimulation in Parkinson's disease. *Movement Disorders*.

[42] Hess C. W. (2013). Modulation of cortical-subcortical networks in Parkinson's disease by applied field effects. *Frontiers in Human Neuroscience*.

[43] Mcintyre C. C., Foutz T. J. (2013). Computational modeling of deep brain stimulation. *Handbook of Clinical Neurology*.

[44] Brocker D. T., Grill W. M. (2013). Principles of electrical stimulation of neural tissue. *Handbook of Clinical Neurology*.

[45] Sutton A. C., Yu W., Calos M. E., Smith A. B., Ramirez-Zamora A., Molho E. S., Pilitsis J. G., Brotchie J. M., Shin D. S. (2013). Deep brain stimulation of the substantia nigra pars reticulata improves forelimb akinesia in the hemiparkinsonian rat. *Journal of Neurophysiology*.

[46] Weiss D., Breit S., Wächter T., Plewnia C., Gharabaghi A., Krüger R. (2011). Combined stimulation of the substantia nigra pars reticulata and the subthalamic nucleus is effective in hypokinetic gait disturbance in Parkinson's disease. *Journal of Neurology*.

[47] Siegel A. (2006). *Essential Neuroscience*.

[48] Stetson D. S., Albers J. W., Silverstein B. A., Wolfe R. A. (1992). Effects of age, sex, and anthropometric factors on nerve conduction measures. *Muscle and Nerve*.

[49] Malmivuo J., Plonsey R. (1995). *Bioelectromagnetism: Principles and Applications of Bioelectric and Biomagnetic Fields*.

